# The trans-DATA study: aims and design of a translational breast cancer prognostic marker identification study

**DOI:** 10.1186/s41512-019-0065-6

**Published:** 2019-10-17

**Authors:** Tim C. de Ruijter, Kim M. Smits, Maureen J. Aarts, Irene E. G. van Hellemond, Leander Van Neste, Bart de Vries, Petronella G. M. Peer, Jürgen Veeck, Manon van Engeland, Vivianne C. G. Tjan-Heijnen

**Affiliations:** 1 Division of Medical Oncology, Maastricht University Medical Center, PO Box 5800, 6202 AZ Maastricht, The Netherlands; 2 GROW – School for Oncology and Developmental Biology, Maastricht University Medical Center, Maastricht, The Netherlands; 3 Department of Pathology, Maastricht University Medical Centre, Maastricht, The Netherlands; 4 Department of Pathology, Zuyderland Medical Centre, Heerlen, The Netherlands; 5 Biostatistics, Department for Health Evidence, Radboud Institute for Health Sciences, Radboud University Medical Center, Nijmegen, The Netherlands

**Keywords:** Breast cancer, Predictive factors, Prognostic factors, Adjuvant treatment, Hormone receptor-positive, Endocrine treatment

## Abstract

**Background:**

The effect of extended adjuvant aromatase inhibition in hormone-positive breast cancer after sequential tamoxifen, aromatase inhibitor treatment of 5 years was recently investigated by the DATA study. This study found no statistically significant effect of prolonged aromatase therapy. However, subgroup analysis showed post hoc statistically significant benefits in certain sub-populations. The trans-DATA study is a translational sub-study aiming to identify DNA methylation markers prognostic of patient outcome.

**Methods:**

Patients from the DATA study are included in the trans-DATA study. Primary breast tumour tissue will be collected, subtyped and used for DNA isolation. A genome-wide DNA methylation discovery assay will be performed on 60 patients that had a distant recurrence and 60 patients that did not have a distant recurrence using the Infinium Methylation EPIC Bead Chip platform. Differentially methylated regions of interest will be selected based on Akaike’s Information Criterion, Gene Ontology Analysis and correlation between methylation and expression levels. Selected candidate genes will subsequently be validated in the remaining patients using qMSP.

**Discussion:**

The trans-DATA study uses a cohort derived from a clinical randomised trial. This study was designed to avoid common pitfalls in marker discovery studies such as selection bias, confounding and lack of reproducibility. In addition to the usual clinical risk factors, the results of this study may identify predictors of high recurrence risk in hormone receptor-positive breast cancer patients treated with sequential tamoxifen and aromatase inhibitor therapy.

## Introduction

Breast cancer is one of the leading causes of cancer-related death for women, although mortality has steadily decreased over the last decade [1, 2]. The improved outcome can be explained by increased early detection in national screening programmes as well as improved systemic treatment options [3]. In the overviews from the Early Breast Cancer Trialist Collaborative Group (EBCTCG) for hormone receptor-positive breast cancer, it was shown that 5 years of adjuvant tamoxifen therapy reduces the risk of breast cancer recurrence by approximately 40% [4]. Treating patients with 5 years of aromatase inhibitor or with 2 to 3 years of tamoxifen followed by aromatase inhibitor (sequential therapy) results in a further reduction of 5-year recurrence rates with 30% compared to tamoxifen alone [5]. However, although much has been achieved, the risk of recurrence persists despite the use of adjuvant endocrine therapy with an annual hazard of recurrence for post-menopausal patients of 2–3% from 10 to 20 years after diagnosis [6].

In daily practice, the risk of recurrence and the need of adjuvant systemic therapy are generally established by considering factors such as nodal status, tumour size, histological grade and/or KI-67, HER2 status and hormone receptor status [7]. For this purpose, nomograms such as the UK-based PREDICT tool [8] or the recently renewed new adjuvant online website may be used to support the shared decision-making process [9].

In recent years, it became evident that biomarkers based on tumour biology may have additional value in predicting patient outcome when combined with parameters that merely reflect anatomical issues [10]. Biomarker (panels), either analysed by immunohistochemistry [11], expression arrays [12], qRT-PCR [13] or nCounter [14] have been developed. Epigenetic alteration, and DNA methylation in particular, have been shown to be common and early events in cancer biology [15, 16]. DNA methylation profiles can be used to identify molecular subclassifications of breast tumours [17–20]. Recent studies have suggested that DNA methylation markers may be of prognostic [18, 21, 22] or predictive [23] value in breast cancer. DNA methylation markers may therefore provide additional information, which may be useful in assessing the risk of recurrence and the most appropriate therapy to administer. In current daily practice, however, no biomarkers have been identified or implemented yet to assess the remaining risk of developing a distant recurrence for the group of patients with hormone receptor-positive disease treated with adjuvant endocrine treatment [24].

The Dutch DATA study is an open-label phase III study, in which we randomly assigned 1912 postmenopausal women with hormone receptor-positive breast cancer after 2–3 years of adjuvant tamoxifen to either 3 or 6 years of anastrozole therapy, with disease-free survival as the primary endpoint. In the DATA study, we observed a post hoc significant benefit from extended use of aromatase inhibitors in the subgroup of patients at increased risk of recurrence based on a positive nodal status and who had both oestrogen receptor (ER)- and progesterone receptor (PR)-positive disease [25]. But, in addition, we observed that many of these patients still developed distant recurrences even though they had used (extended) hormonal treatment, demonstrating the need for new biomarkers that might identify these particular women and that might be used to develop new targeted therapies.

In the trans-DATA study, we will collect paraffin tumour blocks from patients included in the aforementioned DATA study. Here, we present the study protocol of the trans-DATA study, which aims to identify and validate DNA methylation markers that are associated with distant disease recurrence in patients with hormone receptor-positive breast cancer treated with tamoxifen followed by an aromatase inhibitor.

## Patients and methods

### Study objective

The aim of the trans-DATA study is to identify DNA methylation markers associated with the distant recurrence-free interval (DRFI) [26], in patients with hormone receptor-positive disease who underwent adjuvant endocrine treatment within the scope of the DATA trial.

First, we will identify candidate methylation markers in a discovery cohort, irrespective of assigned or delivered adjuvant endocrine treatment duration, by analysing tumour tissue of patients with versus without a distant breast cancer recurrence within the first 6 years after randomization. Next, we will assess whether the identified markers are associated with DRFI in a second, validation cohort. For the validation cohort, the 6-year DRFI will be calculated from the date of randomization in the Dutch DATA study, again irrespective of adjuvant endocrine treatment duration. Events ending a period of DRFI are distant breast cancer recurrences. Patients will be censored in case of death from non-breast cancer causes and at end of follow-up. Local or regional recurrences will not be considered as an event and will also not be a reason for censoring.

### DATA study

For the trans-DATA study, patients are derived from the DATA study (ClinicalTrials.gov; number NCT00301457.), an open-label, multicentre, phase III study, performed in 79 hospitals in the Netherlands [25]. In the DATA study, 1912 postmenopausal women with hormone receptor-positive breast cancer were randomly assigned to 3 years or 6 years of anastrozole therapy after 2 to 3 years of tamoxifen treatment from June 28, 2006, to August 10, 2009. Of these, 1860 are eligible for the primary study endpoint analysis which was defined as disease-free survival starting beyond 3 years after randomisation (adapted disease-free survival). The 5-year adapted disease-free survival was 83.1% in the 6-year and 79.4% in the 3-year group, yielding a hazard ratio of 0.79 (95% CI 0.62 to 1.02). Post hoc exploratory subgroup analysis of patients with oestrogen receptor and progesterone receptor-positive expression having node-positive disease showed a 5-year adapted disease-free survival of 84·4% in the 6-year versus 76·2% in the 3-year group (*n* = 849; HR 0·64 [95% CI 0·46–0·89], *p* = 0·0075).

### Patients

**Fig. 1:**
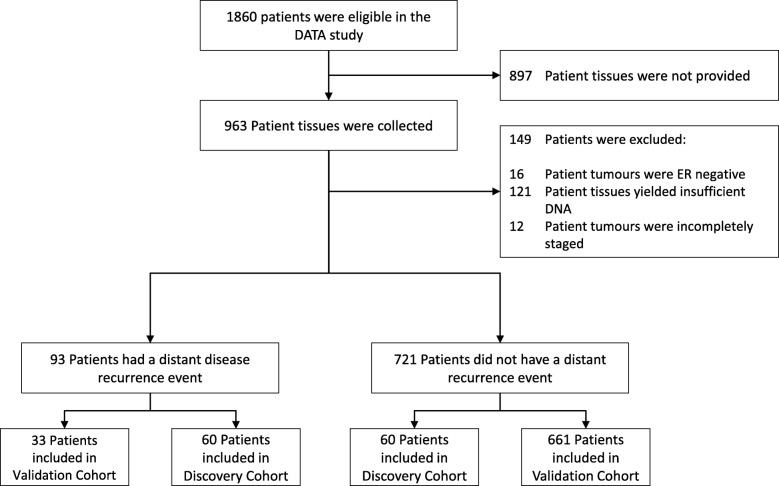
Trans-DATA patient inclusion

For the trans-DATA study, we select patients from the DATA study of whom formalin-fixed paraffin-embedded (FFPE) tissue blocks could be collected (*N* = 963). Patients are excluded when the tumour block was ER negative (*N* = 16), when insufficient tumour tissue is available for DNA extraction (*N* = 121), or when tumour staging is incomplete (*N* = 12). This results in 814 trans-DATA study participants (Fig. 1). Ethical approval for the trans-DATA study was granted by the Medical Ethical committee Arnhem-Nijmegen (MEC registration 2014/02). Samples and patient data used in this study are anonymised and handled according to the Dutch code of conduct for responsible use of human tissue [27, 28].

### Sample work-up

All FFPE tissue blocks will be centrally reviewed by expert pathologists in order to mark areas of representative invasive carcinoma. Subsequently, tissue microarrays (TMAs) will be constructed of all included samples containing three 0.6 mm cores from each tumour sample. Two cores will be taken from the pushing border of the tumour, and one core will be taken from the non-necrotic tumour centre. Areas of ductal carcinoma in situ will be avoided. These TMAs will be used to perform intrinsic subtyping by immunohistochemistry (IHC), according to the 2015 St Gallens consensus [7]. IHC scoring will be performed on 0.5-μm TMA slides. ER, PR and Ki-67 status will be recorded as the percentage of positive tumour cells. If 10% or more of the tumour cells is ER or PR positive, then the tumour is considered hormone receptor-positive. HER2 will be scored as 0, 1+,2+ or 3+, and 2+ cases will be re-evaluated using CISH. HER2 status will be scored as positive if one out of three cores is3+ or CISH amplified. Tumour characteristics such as size, grade and TNM stage will be based on pathology reports. For patients who received neoadjuvant chemotherapy, the clinical T- and N-status at diagnosis were reported if more advanced than the pathologic status. Invasive tumour tissue will be dissected using the haematoxylin and eosin-stained slide as a guide. Next, DNA will be isolated from two–five 20-μm consecutive tumour sections using the Maxwell FFPE CSC automated DNA extraction kit (Promega Corporation, Madison, USA).

### Discovery and validation cohorts

To assure an equal baseline risk for known and routinely used prognostic factors, we will match patients using a propensity risk score approach [29]. The propensity score will be based on age, TN stage, tumour histological grade, hormone receptor status and HER2 status and formulated using logistic regression comparing patients with distant metastases from the DATA study to patients with a 6-year disease-free period. For the discovery cohort, we will match all patients with distant metastasis from the trans-DATA study to disease-free controls from trans-DATA using the developed propensity scores. Thereafter, we will randomly select 60 matched pairs for biomarker discovery analyses.

All patients not included in the discovery cohort will form the validation cohort, used for validation of selected candidate markers.

### Genome-wide DNA methylation analysis

Genome-wide methylation profiles of the discovery cohort will be acquired using the Infinium Methylation EPIC Bead Chip platform (MEBC) (Illumina, San Diego, USA) after DNA restoration (Illumina HD FFPE Restoration kit, Illumina). DNA samples showing fragmentation < 200 base pairs, tested by electrophoresis, will be discarded and replaced. After bisulphite conversion (EZ DNA Methylation Gold Kit, Zymo Research, Irvine, USA), samples undergo whole genome amplification, followed by fragmentation and hybridisation to the Infinium bead chips.

Methylation status for each probe is calculated as a *β* value ranging between 0 and 1, representing the ratio of methylated alleles. A detection *p* value based on the total fluorescence intensity will be calculated for all probes. Probes with a *p* value > 0.01, indicating that overall fluorescent intensity of both the methylated and unmethylated probes does not yield a significantly higher intensity over background, will be excluded from the analysis.

### Marker discovery procedure

To assess differentially methylated promoter regions, methylation profiles of patients with distant recurrent breast cancer will be compared to profiles of patients without distant recurrence. Promoter regions are defined as 1000 bp upstream and 500 bp downstream of each transcript-specific transcription start site as listed in Ensembl GRCh37. If multiple transcripts share an identical set of probes in their promoter region, only one is retained. Only promoter regions overlapping with CpG islands are selected. For statistical analyses, probe *β* values will be transformed into *M* values using the following formula: $M=\log \left(\frac{\beta }{1-\beta}\right)$[30]. A *p* value will be calculated for each individual probe based on a paired, non-parametric test (Wilcoxon rank-sum test). Per promoter region, a single *p* value will be calculated by combining these *p* values for all probes within this promoter region using Fisher’s method [31]. To account for multiple hypothesis testing, a false discovery rate (FDR) method will be applied converting promoter *p* values into *q* values as proposed by Benjamini and Hochberg [32]. Promoter regions with *q* values < 0.001 are considered differentially methylated.

To find promoter regions associated with survival, the prognostic value of each individual probe will be assessed using a Cox’ proportional hazards model. Each probe *M* value will be converted into a binary value indicating methylation or no methylation. Binary cut-off values will be calculated for individual probes based on the optimal sensitivity and specificity to identify patients who developed recurrent breast cancer, by determining the maximum Youden index [33]. Individual probe *p* values will be combined into a single *p* value per promoter region by Fisher’s method and converted into FDR *q* values. Promoter regions with a Cox’ proportional hazards derived *q* value < 0.001 are considered statistically significantly associated with 6-year DRFI. Promoter regions that were both differentially methylated and significantly associated with 6-year DRFI are considered as potential candidate biomarkers.

To further select the most promising markers, Akaike’s Information Criterion (AIC) and Gene ontology analysis will be used. The AIC will be used to identify the combination of potential markers that best predict recurrence, applying a forward selection approach. In addition, a gene ontology analysis will be performed by determining pathways enriched for potential candidate markers according to gene ontology biological process terms and Kyoto Encyclopaedia of Genes and Genomes (KEGG) pathway enrichment analyses using Database for Annotation, Visualization and Integration Discovery (DAVID; https://david.ncifcrf.gov/home.jsp). Pathways are considered statistically significantly enriched at an FDR < 0.1.

**Fig. 2:**
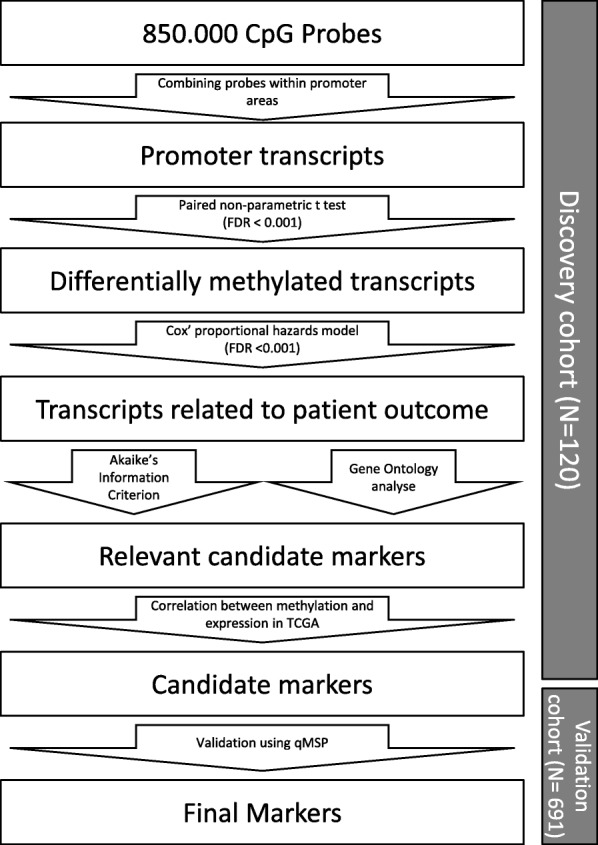
Trans-DATA marker discovery and validation

To assess the area of interest within the promoter region, graphical inspection will be used; this area of interest must contain at least three subsequent differentially methylated probes (*p* < 0.01). Finally, the correlation between DNA methylation in these promoter regions and gene mRNA expression in breast cancer will be assessed using publically available HumanMethylation 450K bead chip and Illumina Hi-seq data downloaded from the TCGA database. A *p* value of 0.05 is chosen as a cut-off for statistical significant correlation between methylation and expression. Only areas of interest in which methylation of all probes is significantly associated with expression will be selected as final markers (Fig. 2).

This discovery method will be performed using three different patient subsets: one including all discovery patients (overall subset *N* = 60 patient pairs), one including patient pairs that underwent recurrence within 3 years (early subset, *N* = 43 patient pairs) and one including patients with time to event longer than 3 years (late subset, *N* = 17 patient pairs.) Markers identified by applying the previously described discovery procedure to these subsets will all be further analysed.

### Validation method

To minimise the risk of false-positive results, a validation analysis of the identified markers in a validation set consisting of all patients that are not part of the MEBC analysis will be done. Quantitative methylation-specific PCR (qMSP) will be used as a validation assay after bisulphite conversion (Epitect 96 wells bisulphite conversion kit, Qiagen, Hilden, Germany). qMSP will be performed using 20 ng of DNA per sample, an in vitro methylated DNA (IVD) standard curve will be included in each reaction to assess absolute methylation levels and a methylation-specific primer set for beta-actin (ACTB) to correct for variation input. A percentage of fully methylated reference (PMR) value will be calculated for each sample and marker according to the following formula: $\frac{Sq\left(\mathrm{marker}\ \mathrm{sample}\right)/ Sq\left(\mathrm{marker}\ \mathrm{IVD}\right)}{Sq\left(\mathrm{ACTB}\ \mathrm{sample}\right)/ Sq\left(\mathrm{ACTB}\ \mathrm{IVD}\right)} \times 100$. PMR values will be interpreted as either methylated or unmethylated using a cut-off value calculated for each marker based on the optimal Youden index in the discovery set. Markers for which no working q-MSP assay can be constructed will be discarded.

Kaplan-Meier curves and log-rank tests are used to estimate the prognostic influence of individual markers. Hazard ratios and corresponding 95% confidence intervals are assessed using a Cox proportional hazard model, adjusted for known prognostic covariates. Possible confounders included in the model are age, pT and pN stage, tumour grade according to modified Bloom Richardson score [34], PR-status and HER2 status in addition to the biomarker. Finally, a Cox proportional hazards model will be developed by including all prognostic clinical factors and q-MSP marker results. We will perform a backward elimination procedure using the likelihood-ratio test to build a multivariate prediction model containing multiple methylation markers; a liberal *α* of 0.10 will be applied to prevent exclusion of potentially important predictors from the model [35]. Model performance will be assessed using Harrell’s C-statistic and AIC. The preferred model is the one with the lowest AIC and the highest C-statistic. To correct for a too optimistic estimation of the C-statistic, the preferred model will be internally validated using bootstrapping (number of bootstraps: 1000) [35]. Results from this validation step will be used to penalise the C-statistic for optimism to prevent too optimistic predictions for future patients.

## Discussion

Patients with breast cancer, in particular patients with hormone receptor-positive disease, remain at risk of distant recurrence for many years after diagnosis. We performed the DATA study to assess whether extended adjuvant aromatase inhibitor therapy duration after the initial 2–3 years of tamoxifen therapy would improve disease-free survival in patients with hormone receptor-positive breast cancer. In the entire study population, we found only a non-significant trend for an improved 5 year adapted disease-free survival of 83% versus 79%, although the follow-up time may be considered as still relatively short. Interestingly, post hoc exploratory subgroup analyses suggest that patients with baseline clinical high-risk disease characteristics may benefit the most from extended adjuvant endocrine therapy. Yet, to date, there is no widely accepted method to estimate the risk of distant recurrence after initial adjuvant endocrine treatment in breast cancer. In the trans-DATA study, we aim to identify DNA methylation biomarkers predictive of distant recurrence and design a model including these biomarkers and routinely used clinical factors, to identify patients with hormone receptor-positive breast cancer with an increased long-term risk of distant recurrence.

Only 0.8% of all published cancer DNA methylation biomarkers have been translated into clinical applications for the management of cancer [36], and none have been developed for diagnosis and prediction of prognosis and response to therapy in breast cancer. Failure of previously identified DNA methylation markers to achieve clinical utility can be explained by many factors, e.g. poor identification of a particular study population, inadequate reporting of translational research methods and results and inadequate marker validation [37–40]. These factors may result in poor reproducibility of identified markers. The trans-DATA study is based on the clinical phase III DATA trial population, with well-described handling of differences in baseline characteristics, and use of Fisher’s test to analyse promoter methylation status, which may help to overcome some of these pitfalls.

Cohorts derived from randomised control trials such as the DATA study provide a unique opportunity for biomarker research. These cohorts are homogenously treated and include prospectively collected clinical follow-up, limiting the influence of misclassification bias [40]. Recent translational results from the clinical phase III ATAC and TEAM trials have shown the potential of translational research projects from these randomised control trial-derived cohorts [13, 41]. For example, the patient cohort included in the ATAC trial was employed to study the prognostic value of biomarker assays such as the Oncotype DX [13] and Breast Cancer Index [24], and results from these translational studies were subsequently included in the American Society of Clinical Oncology practical guideline on the use of biomarkers for clinical decision making in breast cancer treatment [42].

Marker performance is also influenced by the selection of an appropriate endpoint [38]. As prevention of distant breast cancer recurrence is the main goal of adjuvant endocrine therapy in patients with hormone receptor-positive disease, distant recurrence free-interval should in our opinion be the primary study objective of translational biomarker studies aiming at identifying patients who might benefit from additional adjuvant therapies. We chose to assess the 6-year DRFI as the primary endpoint as this was the maximum treatment duration of adjuvant endocrine therapy allowed within the framework of the DATA trial. Of note, all patients had already received 2 to 3 years of tamoxifen before randomisation, but in the TRANS-DATA trial, we calculated DRFI from date of randomisation. Hence, we actually look at patients who had a diagnosis of breast cancer 8 to 9 years prior to the primary endpoint. Nevertheless, we know that even though this is already a long period of observation, patients with hormone receptor-positive disease may have recurrences for many more years. Therefore, after the first 6-year DRFI analysis, we additionally aim to assess the occurrence of distant recurrences until the year 2022, and we will report on the follow-up results. By doing so, we will assess whether the identified markers will still hold their relevance, even after a very long period of observation, which may then strengthen our results even more.

Using propensity score matching, we chose to match the discovery cohort patients for parameters currently used by clinicians to assess the risk of recurrence in breast cancer [9], to minimise differences in baseline characteristics between the recurrent and non-recurrent group. These differences could lead to the identification of candidate genes related to baseline characteristics rather than disease outcome. Propensity score matching is especially appropriate when selecting patients from a cohort consisting of a small portion of event subjects and a large portion of non-event subjects, as is the case in our discovery analysis [43].

Assaying genome-wide DNA methylation data at single CpG resolution is of limited value and is hampered by the need for extensive multi-hypothesis correction [44, 45]. Due to the vast number of CpGs on the MEBC array, only the strongest single CpG differences remain significant, resulting in false-negative results, especially for small effect sizes [44]. Differential methylation is often not restricted to a single CpG site but may span CpGs over several hundreds of base pairs [46]. We will therefore analyse differential methylation by combining measurements across promoter regions employing Fisher’s combined probability test as previously described by Assenov et al. [31]. This method facilitates the identification of differentially methylated areas because it is capable of identifying grouped CpGs with small methylation differences [31]. In addition, by analysing the data per promoter instead of per CpG, we decrease the number of hypotheses we need to correct for and focus on biologically and clinically relevant biomarkers. Genome-wide discovery assays are inherently noisy and imprecise and therefore prone to false-positive results [38, 47]. To minimise the number of false discoveries, candidate genes must fit a stringent set of requirements in our analyses. Candidate markers must show both differential methylation and a significant relation to the risk of recurrence. Moreover, we will only include markers that show a significant correlation between DNA methylation and gene expression in breast cancer data derived from the TCGA database, further ensuring a possible biological relevance of the candidate markers. Finally, we include a validation of all candidate markers in a separate subset of patients. We expect that this stringent approach will result in biomarkers with a high chance of reproducibility.

Despite our attempts to overcome known problems in prognostic studies, our study has some limitations. First, like all array-based techniques, findings by MEBC are biased towards probe availability on the chip. It is therefore possible that potential markers will be missed simply because no probes are located near these markers. Second, even though there is no overlap between the discovery and the validation cohort, both subgroups are derived from the same cohort. Markers should be further validated in an independent cohort to confirm their association with breast cancer outcome. Further, in order to differentiate between a prognostic and a predictive marker, the performance of the marker has to be assessed in a systemically untreated as well as a treated cohort. Prognostic markers identify patients at risk of relapse after local treatment with curative intent, and predictive markers identify patients that might benefit from a particular (targeted) systemic treatment. In the DATA trial, all patients received endocrine treatment, either randomised for 3 or for 6 years of anastrozole therapy after 2 to 3 years of tamoxifen therapy. As all patients have received the same endocrine treatment during the first 3 years after randomisation, the markers that are identified to be associated with outcome can be either of prognostic or predictive value.

We also recognise that the validation section as proposed in this protocol paper does not provide a complete validation of the selected markers as this analysis is used to further condense these markers into a model including clinical features. In addition, the number of cases in our validation cohort is too small to draw definitive conclusions. We consider our validation analysis a first step in assessing the prognostic value of the marker model but recognise that any model that is provided by this study will need further independent validation.

Despite these limitations, the trans-DATA study has the potential to identify DNA methylation biomarkers for risk of recurrence after adjuvant endocrine treatment. These markers may be combined with routinely used prognostic factors in a model capable of identifying patients that benefit from prolonged adjuvant endocrine treatment or other targeted adjuvant treatments.

## Data Availability

Not applicable

## Abbreviations

ACTB: Beta-actin
AIC: Akaike’s information criterion
ER: Oestrogen receptor
FFPE: Formalin-fixed paraffin-embedded
IHC: Immunohistochemistry
IVD: In vitro methylated DNA
KEGG: Kyoto Encyclopaedia of Genes and Genomes
MEBC: Infinium Methylation EPIC Bead Chip platform
PMR: Percentage of fully methylated reference
PR: Progesterone receptor
qMSP: Quantitative methylation-specific PCR
TMA: Tissue microarray
